# Recent advances in UV-B signalling: interaction of proteins with the UVR8 photoreceptor

**DOI:** 10.1093/jxb/erae132

**Published:** 2024-03-25

**Authors:** Wei Liu, Gareth I Jenkins

**Affiliations:** School of Molecular Biosciences, College of Medical, Veterinary and Life Sciences, Bower Building, University of Glasgow, Glasgow G12 8QQ, UK; School of Molecular Biosciences, College of Medical, Veterinary and Life Sciences, Bower Building, University of Glasgow, Glasgow G12 8QQ, UK; Indian Institute of Science Education and Research, India

**Keywords:** COP1, photomorphogenesis, photoreceptor, protein interaction, RUP protein, UV-B, UVR8

## Abstract

The UV RESISTANCE LOCUS 8 (UVR8) photoreceptor mediates many plant responses to UV-B and short wavelength UV-A light. UVR8 functions through interactions with other proteins which lead to extensive changes in gene expression. Interactions with particular proteins determine the nature of the response to UV-B. It is therefore important to understand the molecular basis of these interactions: how are different proteins able to bind to UVR8 and how is differential binding regulated? This concise review highlights recent developments in addressing these questions. Key advances are discussed with regard to: identification of proteins that interact with UVR8; the mechanism of UVR8 accumulation in the nucleus; the photoactivation of UVR8 monomer; the structural basis of interaction between UVR8 and CONSTITUTIVELY PHOTOMORPHOGENIC 1 (COP1) and REPRESSOR OF UV-B PHOTOMORPHOGENESIS (RUP) proteins; and the role of UVR8 phosphorylation in modulating interactions and responses to UV-B. Nevertheless, much remains to be understood, and the need to extend future research to the growing list of interactors is emphasized.

## Teaser

This short review highlights recent advances in understanding interactions between the UVR8 photoreceptor and proteins involved in regulating gene expression in response to UV-B exposure.

## Introduction

UV-B radiation in sunlight has wide-ranging effects on the biosphere (Barnes *et al.*, 2022). Since UV-B is the most energetic component of the daylight spectrum, it has the potential to damage macromolecules such as DNA and generate reactive oxygen species that are detrimental to normal cell function. Plants protect themselves against the damaging effects of UV-B radiation through acclimatory responses (Jenkins, 2017; Podolec *et al.*, 2021a; Shi and Liu, 2021; Chen *et al.*, 2022). They sense the ambient level of UV-B and initiate changes in their metabolism, development, and physiology to optimize their growth and viability.

Many regulatory responses of plants to UV-B and short wavelength UV-A exposure are mediated by the UV RESISTANCE LOCUS 8 (UVR8) photoreceptor (Jenkins, 2017; Podolec *et al.*, 2021a; Shi and Liu, 2021; Chen *et al.*, 2022). Characterization of Arabidopsis *uvr8* mutant plants has identified numerous biochemical, morphological, developmental, and physiological responses mediated by UVR8 (Jenkins, 2017; Podolec *et al.*, 2021a). For example, UV-B exposure, detected by UVR8, stimulates the biosynthesis of phenylpropanoid compounds that protect against damaging levels of UV radiation (Kliebenstein *et al.*, 2002; Morales *et al.*, 2013) and suppresses extension growth, producing compact plants (Favory *et al.*, 2009; Hayes *et al.*, 2014). Transcriptomic analyses of wild-type and *uvr8* mutant Arabidopsis plants (Brown *et al.*, 2005; Favory *et al.*, 2009; Morales *et al.*, 2013), and plants with UVR8 targeted to the nucleus (Qian *et al.*, 2020), have identified several hundred genes that are differentially expressed following UV-B exposure and which underpin downstream responses initiated by UVR8 signalling.

UVR8 is a seven-bladed β-propellor protein that exists as a dimer in the absence of UV-B (Rizzini *et al.*, 2011; Christie *et al.*, 2012; Wu *et al.*, 2012). The dimer is maintained by a network of salt bridges between charged amino acids on the dimerization surfaces of adjacent monomers. UV-B is absorbed by tryptophan amino acids that channel excitation to specific tryptophans at the dimer interface, leading to neutralization of the salt bridges that hold the dimer together (Christie *et al.*, 2012; Wu *et al.*, 2012; Li *et al.*, 2022). Thus, photoreception causes dimer dissociation, generating monomeric UVR8, which initiates signalling. As discussed below, following photoreception, a small fraction of cellular UVR8 accumulates in the nucleus (Kaiserli and Jenkins, 2007; Qian *et al.*, 2016; Fang *et al.*, 2022) where it is able to regulate gene expression. Present understanding is that only nuclear UVR8 is active, so the role of the cytosolic pool remains unclear. The UVR8 system is dynamic. The continuous formation of monomers under UV-B exposure is balanced by re-association to form dimers, which is mediated by direct interaction with REPRESSOR OF UV-B PHOTOMORPHOGENESIS (RUP) proteins (Heijde and Ulm, 2013). Under constant UV-B exposure in photoperiodic conditions, a photo-equilibrium is established where ~25% of UVR8 is maintained in the monomeric form (Findlay and Jenkins, 2016; Liao *et al.*, 2020).

Recent research has established that UVR8 functions through interactions with other proteins. In addition to the RUP proteins, UVR8 interacts directly with several transcription factors to modify their ability to bind to promoters of target genes involved in responses to UV-B (see below). UVR8 also binds the CONSTITUTIVELY PHOTOMORPHOGENIC 1 (COP1) protein, which associates with a SUPPRESSOR OF PHYA-105 (SPA) protein to form a substrate receptor for an E3 ubiquitin ligase complex (Favory *et al.*, 2009; Huang *et al.*, 2013). This complex degrades a number of proteins, notably the ELONGATED HYPOCOTYL 5 (HY5) transcription factor, which regulates many gene targets of UVR8 signalling. In addition, COP1 binding to UVR8 de-stabilizes PHYTOCHROME INTERACTING PROTEIN 5 (PIF5), which contributes to UV-B suppression of extension growth (Sharma *et al.*, 2019). Hence, interactions with different proteins are crucial in orchestrating UVR8 activity; binding of specific transcription factors in particular cells and tissues will underpin responses to UV-B. It is therefore essential to understand how different proteins are able to bind to UVR8 and how differential binding is regulated. Recent advances in addressing these questions (summarized in Box 1) are the principal focus of this review.

**Box 1. Recent key developments in understanding UVR8 interactions with proteins**

The following key advances are numbered with reference to the schematic below.

1. **A better understanding of UVR8 accumulation in the nucleus**
2. UVR8 interacts with proteins in the nucleus to regulate gene expression. Fang *et al.* (2022) report that UVR8 accumulates in the nucleus through retention resulting from interaction with COP1. RUP proteins inhibit this interaction and promote nuclear export.
3. **New information on photoactivation of the UVR8 monomer**
4. Wang *et al.* (2022) identify structural changes associated with the activated monomer. Podolec *et al.* (2021b) characterize the G101S mutant, which is hyper-responsive to UV-B due to impaired RUP-mediated re-dimerization, and show how the mutation alters structure at the dimerization surface.
5. **Structural information on the interaction of COP1 with UVR8**
6. Wang *et al.* (2022) report the cryo-EM structure of UVR8 bound to COP1, giving detailed information on interfaces with the UVR8 C27 region and with the UVR8 dimerization surface.
7. **Structural information on the interaction of UVR8 with RUP proteins**
8. Wang *et al.* (2023) present a crystal structure for UVR8 bound to RUP2, showing that the molecular basis of interaction is similar to that for UVR8 and COP1.
9. **Phosphorylation of UVR8 modulates protein interaction**
10. Liu *et al.* (2024) report that UVR8 is phosphorylated and that mimicking phosphorylation of Ser402 enhances RUP binding and increases UVR8-mediated flavonoid and hydroxycinnamic acid accumulation.
11. **Binding to UVR8 modifies activity of a DNA methyltransferase**
12. Jiang *et al.* (2021) show that UVR8 binds the DRM2 *de novo* DNA methyltransferase, which modifies its activity and chromatin association, hence modifying DNA methylation in UV-B.

In the schematic, a fraction of the UVR8 monomer produced by UV-B photoreception in the cytosol accumulates in the nucleus where, in the activated conformation (indicated by a change in shape), it interacts with other proteins (see Boxes 2 and 3), modifying their ability to regulate gene expression and hence promoting responses to UV-B. Whether photoactivation occurs in the nucleus (as drawn) or the cytosol is not known. When COP1/SPA and RUP proteins are not bound to UVR8, they form part of an E3 ubiquitin-ligase complex that targets HY5 and other proteins for proteolysis.

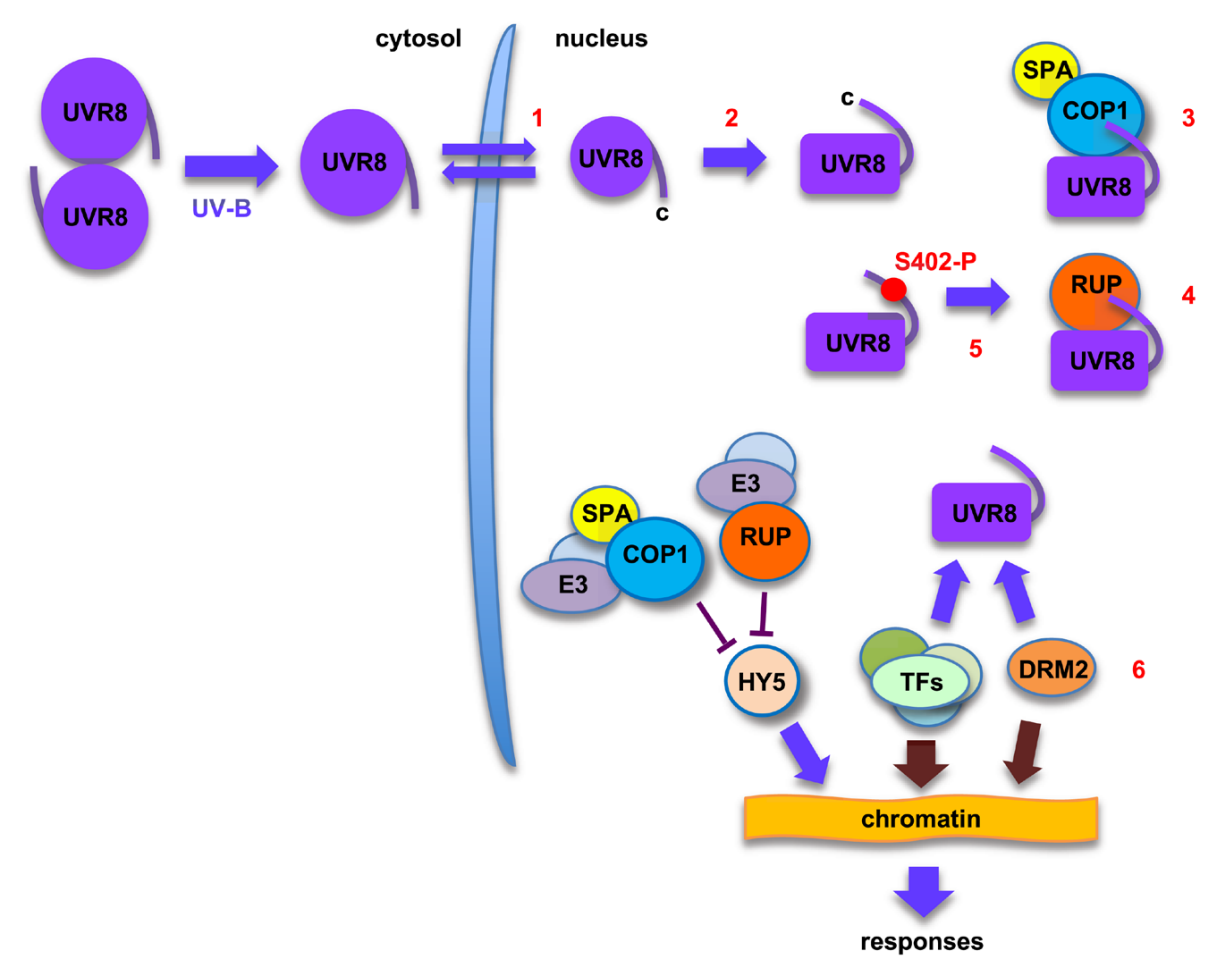

## The list of proteins interacting with UVR8 continues to grow

Interaction of UVR8 with COP1 and RUP proteins was first reported over a decade ago (Favory *et al.*, 2009; Gruber *et al.*, 2010). Direct interaction with transcription factors was first demonstrated in 2018, for the WRKY36 protein, which represses *HY5* transcription (Yang *et al.*, 2018), and for BES1-INTERACTING MYC-LIKE 1 (BIM1) and dephosphorylated BRI1-EMS-SUPPRESSOR 1 (BES1), which regulate gene targets of brassinosteroid signalling involved in extension growth (Liang *et al.*, 2018). Subsequently, several MYB DOMAIN PROTEIN (MYB) transcription factors were shown to bind to UVR8, modifying their activity in promoter binding and impacting various responses (Qian *et al.*, 2020; Yang *et al.*, 2020) (Box 2).

An additional type of protein recently shown to physically interact with UVR8 is a DNA methyltransferase (Jiang *et al.*, 2021) (Box 2). This research revealed that UV-B, acting through UVR8, impairs DNA methylation mediated by DOMAINS REARRANGED METHYLTRANSFERASE 2 (DRM2). This is a key enzyme as it catalyses *de novo* DNA methylation independent of the sequence context. The authors showed that binding to UVR8 inhibits the DNA methyltransferase activity of DRM2 and reduces its association with chromatin. This study highlights the importance of UV-B in modulating DNA methylation in the genome and the role of UVR8 in this process.

**Box 2. Proteins binding to UVR8**

**Table:** 

Protein	Function in UV-B signalling	Reference
COP1	COP1 bound to a SPA protein is the substrate receptor for an E3 ubiquitin ligase that targets HY5 for proteolysis; inhibited by COP1 binding to UVR8. COP1 stabilizes PIF5; inhibited by COP1 binding to UVR8.	Favory *et al.* (2009) Huang *et al.* (2013) Sharma *et al.* (2019)
RUP1/RUP2	Negative regulators of UV-B signalling. Promote UVR8 re-dimerization. Substrate receptors for an E3 ubiquitin ligase that targets HY5 for proteolysis; inhibited by binding to UVR8. RUP2 involved in regulation of flowering by UV-B.	Gruber *et al.* (2010) Heijde and Ulm (2013) Ren *et al.* (2019) Arongaus *et al.* (2018)
WRKY36	Promotes hypocotyl elongation by binding to the *HY5* promoter to inhibit its transcription. Interaction with UVR8 releases the inhibition of HY5.	Yang *et al.* (2018)
BIM1/dephosphorylated BES1	Regulate transcription of target genes of brassinosteroid signalling, which promote hypocotyl elongation. Interaction with UVR8 represses their DNA binding activity.	Liang *et al.* (2018)
MYB73/MYB77	Promote transcription of auxin-responsive genes. Interaction with UVR8 represses DNA binding activity and inhibits lateral root growth.	Yang *et al.* (2020)
MYB13	Regulates expression of genes involved in auxin response and flavonoid biosynthesis. Promotes UV-B-induced cotyledon expansion and UV stress acclimation. Binding to UVR8 differentially modulates transcription of target genes.	Qian *et al.* (2020)
DRM2	*De novo* DNA methylation. Interaction with UVR8 inhibits its DNA methyltransferase activity and reduces its association with chromatin.	Jiang *et al.* (2021)



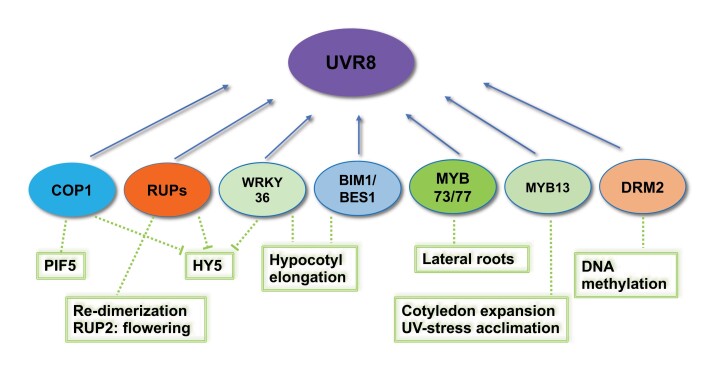

It is very likely that the list of proteins interacting with UVR8 will continue to grow, and these various interactions will underpin the numerous responses to UV-B mediated by the photoreceptor.

## Nuclear localization of UVR8

Localization will determine protein interactions with UVR8. Proteins that interact with UVR8 will not necessarily be present in all cells at all times, as they may be subject to differential expression, for instance in particular tissues or in response to light. For example, *MYB13* expression is strongly stimulated by UV-B exposure and is greater in cotyledons than in hypocotyls (Qian *et al.*, 2020).

A key factor determining interaction is intracellular localization. Transcription factors and DRM2 are localized in the nucleus and will interact with UVR8 following its UV-B-stimulated nuclear accumulation. Recent research by Fang *et al.* (2022) has provided new insights into the mechanism of UVR8 nuclear accumulation. The increased nuclear abundance of UVR8 following UV-B exposure could be explained either by increased translocation into the nucleus or by decreased export. It was previously reported that COP1 is required for UV-B stimulation of UVR8 nuclear accumulation, raising the possibility of a co-import mechanism employing the nuclear localization signal of COP1 (Yin *et al.*, 2016). However, direct measurements of nuclear translocation using a fluorescence recovery after photobleaching (FRAP) method show that the UV-B stimulation of UVR8 translocation is independent of COP1 (Fang *et al.*, 2022). Addition of a triple yellow fluorescent protein (YFP) tag to UVR8, making a fusion protein of at least 130 kDa, impairs nuclear accumulation, suggesting that the protein enters via free diffusion. Using lines in which COP1 was fused to either a nuclear localization signal or a nuclear exclusion signal, the authors showed that nuclear-localized COP1 is required for UV-B-stimulated UVR8 nuclear accumulation. Moreover, interaction between COP1 and UVR8 in the nucleus is required for nuclear accumulation of UVR8. Together the results indicate that UVR8 is retained in the nucleus under UV-B exposure as a consequence of binding to COP1. RUP proteins inhibit the COP1–UVR8 interaction, and thereby facilitate the nuclear exit of UVR8.

The localization of UVR8 within the nucleus remains to be determined. It is likely that it integrates into one or more protein complexes that may include COP1, RUP proteins, various transcription factors, or DRM2, but the mechanisms regulating complex formation are unknown, and it is unclear whether such complexes are associated with chromatin.

## Binding sites for protein interactions with UVR8

Proteins will interact with UVR8 via binding sites with a particular surface topography, hydrophobicity, or charge. A 27 amino acid region within the C-terminal domain of UVR8 (termed C27; amino acids 397–423) was shown to be important in binding both COP1 and RUP proteins (Cloix *et al.*, 2012). A VP motif (V410 and P411) in the C27 region is required for binding to COP1 (Yin *et al.*, 2015; Lau *et al.*, 2019). COP1 was found additionally to interact with the β-propellor region of UVR8 (Yin *et al.*, 2015). However, until recently, the lack of structural information for the flexible, disordered C-terminal domain of UVR8 hampered understanding of the molecular basis of the interaction with COP1. A recent study by Wang *et al.* (2022) has provided valuable new insights into this interaction. They produced a complex of UVR8 bound to COP1 *in vitro* using heterologously expressed proteins. In this complex, COP1 was bound to a truncated SPA protein (SPA4^1–464^). The complex was then examined by cryo-EM. UVR8 was found to bind to COP1 through two interfaces, consistent with previous research (Yin *et al.*, 2015). One of the interfaces is with the C27 region, and includes the VP motif. Mutation of this motif to alanine reduced binding. The second interface involves amino acids on the dimerization surface of UVR8, indicating that COP1 binds to monomeric UVR8 via charged amino acids, similar to the molecular interactions that maintain the UVR8 dimer. Mutation of specific amino acids in the UVR8 dimerization surface impaired binding of COP1. The structural analysis, together with mutation, also identified amino acids of COP1 involved in the interaction.

A similar approach was used by Wang *et al.* (2023) to examine the molecular basis of the interaction between UVR8 and RUP proteins. RUP1 and RUP2 expressed and purified from mammalian cells were able to stimulate re-dimerization of UVR8 monomers *in vitro*. The dynamic nature of the RUP–UVR8 interaction and monomer/dimer conversion prevented the formation of a stable complex for structural studies. Therefore, a constitutively active UVR8 mutant (UVR8^W285A^; Heijde *et al.*, 2013; Huang *et al.*, 2014), which binds to RUP proteins independently of UV-B, was used for complex formation. A crystal structure was obtained for the RUP2–UVR8^W285A^ complex (RUP2 amino acids 21–366; UVR8^W285A^ amino acids 12–421). The structure revealed that RUP2 binds to UVR8 via two interfaces, similar to COP1. One interface is with the C27 region, involving residues 400–413, which is again dependent on the VP motif. The second interface is with the UVR8 dimerization surface, via charged amino acids involved in UVR8 dimerization, similar to the interaction between UVR8 and COP1. Further experimentation showed that plants with mutations in RUP2 amino acids critical in the interaction with UVR8 are deficient in UVR8 re-dimerization and in UVR8-mediated photomorphogenesis.

Despite the above important advances, it remains unclear why the interaction with COP1 only occurs with the monomer, whereas RUP proteins can also interact with the dimer in conditions without UV-B, albeit to a lesser extent (Cloix *et al.*, 2012; Yin *et al.*, 2015; Wang *et al.*, 2023; Liu *et al.*, 2024). In addition, little is known about the interaction of UVR8 with other proteins, except in some cases there is evidence that the β-propeller core, C-terminal 44 amino acids, or C27 region is involved (Box 3).

**Box 3. Binding sites of UVR8–protein interactions**

**Table:** 

Protein	Binding sites of UVR8	Binding sites of interacting protein	Reference
COP1	β-Propellor core dimerization surface; C27 region, V410, and P411	WD40; specific amino acids identified	Favory *et al.* (2009) Rizzini *et al.* (2011) Cloix *et al.* (2012) Yin *et al.* (2015) Lau *et al.* (2019) Wang *et al.* (2022)
RUP1/RUP2	β-Propellor core dimerization surface; C27 region, V410, and P411	WD40; specific amino acids identified	Cloix *et al.* (2012) Yin *et al.* (2015) Wang *et al.* (2023)
WRKY36	C44 region (397–440)	C-terminal DNA-binding domain (191–388)	Yang *et al.* (2018)
BIM1/de-phosphorylated BES1	C44 region (397–440)	BIM1: C-terminus alone, or bHLH with either N- or C-terminal domains; BES1: BIN2 phosphorylation domain	Liang *et al.* (2018)
MYB73/MYB77	β-Propellor core; C44 region (397–440)	MYB73 and MYB77: N-terminal R2R3 DNA-binding domain 1–120	Yang *et al.* (2020)
MYB13	Unknown	Unknown	Qian *et al.* (2020)
DRM2	β-Propellor core; C44 region (397–440)	UBA domain	Jiang *et al.* (2021)



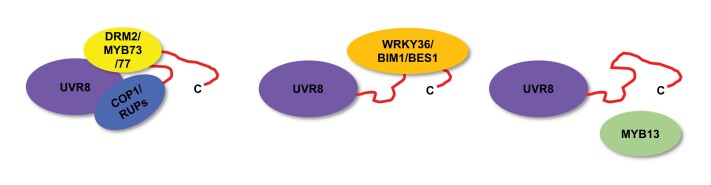

## Interaction will be dependent on binding affinity, amount of interactors, and UVR8 conformation

Whether particular proteins bind to UVR8 will be dependent on several factors: their relative binding affinities, the relative amounts of interactors, and whether the binding sites are accessible.

A notable conclusion from the structural studies is that RUP2 and COP1 interact with UVR8 through very similar molecular mechanisms. Moreover, there is evidence that RUPs outcompete COP1 for binding to UVR8 *in vitro* (Wang *et al.*, 2022), indicating that they have a higher binding affinity. This finding supports the concept that RUP proteins displace COP1 to promote UVR8 re-dimerization. However, there are few quantitative data about the relative abundance of COP1 and RUP proteins in the cytosol and nucleus, so information on how these proteins compete to bind UVR8 *in vivo* is incomplete. Moreover, there is no information on binding affinities and relative amounts of other proteins that interact with UVR8. Whether multiple proteins compete for binding to C27, or whether there could be co-operative binding is not yet known.

There is evidence that UV-B photoreception initiates conformational changes to the monomer (Rizzini *et al.*, 2011; Heilmann *et al.*, 2015; Miyamori *et al.*, 2015; Zeng *et al.*, 2015; Camacho *et al.*, 2019). Conformation will determine which binding sites are presented and hence which proteins can bind. Most studies of conformation have been undertaken with purified UVR8 protein and, in some cases, quite dramatic changes, including partial unravelling of the β-propellor core, have been reported (Zeng *et al.*, 2015; Camacho *et al.*, 2019). Whether such extensive changes occur *in vivo*, where UVR8 will usually be bound to other proteins, is unknown. One study, using fluorescence resonance energy transfer (FRET) to examine UVR8 conformation *in vivo* (Liao *et al.*, 2019), found little evidence of conformational change.

Formation of the UVR8 monomer is insufficient on its own to initiate signalling; the monomer needs to acquire an ‘activated’ conformation (Jenkins, 2014). Thus, UV-B-induced dimer dissociation and UV-B photoactivation of the monomer can be considered as distinct processes. To illustrate, the UVR8^D96N,D107N^ mutant (Heilmann *et al.*, 2016) is constitutively monomeric *in vivo*, but requires UV-B exposure to initiate responses. Whether photoactivation occurs in the cytosol or the nucleus and whether it involves monomer photoreception (Heilmann *et al.*, 2016) are unknown. Recent research has provided new information on the activated state and on amino acids involved in making a signalling-active conformation. Wang *et al.* (2022) observed structural changes following UV-B activation of UVR8, notably alterations to the orientation of tryptophans in the dimer interface involved in photoreception and their adjacent charged amino acids. Podolec *et al.* (2021b) identified a semi-dominant mutant, UVR8^G101S^, which is monomeric *in vivo* but requires UV-B for activation. The mutant exhibits a hyper-response to UV-B, and overexpression of UVR8^G101S^ results in an exaggerated photomorphogenesis phenotype. UV-B-induced COP1 binding is not increased in the mutant, and the enhanced responsiveness is due to impaired re-dimerization by RUP1 and RUP2. Plants expressing the combination of mutations UVR8^G101S^ and UVR8^W285A^ have a very strong constitutive photomorphogenesis phenotype. The crystal structure of UVR8^G101S,W285A^ shows that the G101S mutation distorts a critical loop in the dimerization interface that will impact the ability of RUP proteins to promote dimer formation (Podolec *et al.*, 2021b).

As outlined above, structural studies combined with mutant characterization are providing insights into how UV-B photoreception causes UVR8 monomerization and monomer activation. This research can be extended to examine how conformational changes in UVR8 enable the photoreceptor to interact with additional proteins.

## Phosphorylation modulates protein interactions with UVR8

Post-translational modification is an important potential mechanism for regulating interactions of proteins with UVR8. However, until recently, there was no evidence, other than from proteomic databases, that UVR8 is subject to modification *in vivo*. Liu *et al.* (2024) labelled Arabidopsis seedlings with ^32^P and showed that immunoprecipitated UVR8 was phosphorylated. Moreover, there was an increase in labelling within 6 h of exposure to UV-B. MS revealed that relatively few sites were consistently phosphorylated. Labelling of mutants showed that the C27 region is the main site of phosphorylation and that Ser402 is the principal phosphorylated amino acid within C27. An antibody was produced that recognizes the Ser402-phosphorylated form of UVR8, which shows a slight difference in electrophoretic mobility, consistent with the altered charge. UVR8 phosphorylated at Ser402 is only a small fraction of the total UVR8 population, as it cannot be detected by standard anti-UVR8 antibodies. Use of the phospho-antibody demonstrated that Ser402 phosphorylation is stimulated following UV-B exposure, and examination of nuclear and cytosolic fractions showed that Ser402 phosphorylation increases in the nucleus. Together, the results suggest that Ser402 phosphorylation is enhanced in the nucleus as a result of UV-B-stimulated nuclear accumulation. It was further shown that SPA1 protein kinase activity is required for Ser402 phosphorylation, although it is not yet clear whether SPA directly phosphorylates UVR8.

The role of Ser402 phosphorylation in regulating protein interactions was investigated using mutants: UVR8^S402A^, which is unable to be phosphorylated, and UVR8^S402D^, which is a phosphomimetic mutation due to the charge introduced by the aspartate residue. Liu *et al.* (2024) found that the mutants were unaltered in binding to COP1 and the WRKY36 transcription factor, but UVR8^S402D^ showed strongly increased binding to RUP proteins. UVR8^S402A^ showed relatively little alteration in RUP binding, but this may be due to the dual binding site of the RUP proteins; although the S402A mutation may eliminate binding to C27, RUPs are still able to bind via the β-propellor region of UVR8. Together the data suggest that Ser402 phosphorylation may be important in modulating protein interactions with UVR8, evidenced by the enhanced binding of RUP proteins to UVR8^S402D^.

The effect of Ser402 phosphorylation on responses mediated by UVR8 was examined using plants expressing the Ser402 mutants (Liu *et al.*, 2024). UVR8^S402D^ plants growing under a low level of UV-B exposure (UV-B-acclimated plants) showed increased accumulation of HY5 protein. There was no corresponding change in *HY5* transcript abundance, indicating that elevated HY5 expression is most probably due to increased stability of the protein. HY5 is known to be targeted for proteolysis by E3 ubiquitin ligases involving COP1–SPA (Favory *et al.*, 2009; Huang *et al.*, 2013) or RUP proteins (Ren *et al.*, 2019). Since UVR8^S402D^ is not altered in COP1 binding but has increased RUP binding, the authors suggested that HY5 may be stabilized by decreased availability of the RUP–E3 complex. Accumulation of CHALCONE SYNTHASE (CHS), the first enzyme in the flavonoid biosynthesis pathway, was also increased in UVR8^S402D^ plants. Again, the protein showed increased stability but the mechanism involved in stabilizing CHS in UVR8^S402D^ plants is not clear. Nevertheless, the UVR8^S402D^ plants had increased accumulation of flavonoids and hydroxycinnamic acids in response to UV-B exposure. This research provides evidence that Ser402 phosphorylation modulates particular UVR8-mediated responses, although further studies are needed to determine whether it affects other responses. Additionally, further research is needed to examine the possible significance of phosphorylation at other sites.

## Conclusions and perspective

The recent advances highlighted above have provided valuable new insights into UVR8 function: a better understanding of nuclear accumulation, structural details of the binding sites for COP1 and RUP2, new information on monomer activation, and the discovery that UVR8 is phosphorylated and that phosphorylation modulates particular protein interactions and downstream responses. However, numerous important questions remain to be answered. For example, it is still unknown why most UVR8 is present in the cytosol—does cytosolic UVR8 have a specific function? Information is also lacking on the sequence of events that occur following the appearance of UVR8 in the nucleus: monomer activation, phosphorylation, protein interactions, and probably association with protein complexes; and, furthermore, on the mechanisms of protein dissociation and nuclear exit, which is facilitated by RUP proteins (Fang *et al.*, 2022). In addition, we do not understand how RUP proteins act to promote UVR8 re-dimerization—the indication that RUP proteins have a higher affinity for UVR8 monomer than COP1 (Wang *et al.*, 2022) does not explain the process of UVR8 monomer re-association, which entails the dissociation of RUPs from the dimerization surface of UVR8.

The discovery, in recent years, that UVR8 interacts with multiple proteins to direct responses to UV-B has been a major step forward in understanding how the photoreceptor functions. Moreover, it is likely that additional interactors that enable UVR8 to mediate specific responses to UV-B will be identified. However, while excellent progress has clearly been made, the identification of multiple interactors has raised challenging questions in UVR8 research: how does the photoreceptor bind to these different proteins and what are the molecular mechanisms that control differential binding? These are crucial questions because the proteins that bind to UVR8 in a particular cell at a particular time will determine the direction of the response or, in the case of RUP proteins, the likelihood of re-dimerization.

The challenge now is to understand how the growing list of interacting proteins function with UVR8. What are their binding sites and binding affinities—do they compete for binding at the same site as COP1 and RUP proteins or bind elsewhere on the protein? Interaction assays and structural studies will be needed to address these questions. The regulation of differential binding also needs to be investigated—do conformational changes associated with monomer activation differentially affect binding? To what extent does phosphorylation or other post-translational modifications determine which proteins will bind? The effects of specific modifications on different interactions will need to be examined. Nevertheless, given the recent rate of progress, further important advances in understanding UVR8 function can be expected in the near future.

## References

[CIT0001] Arongaus AB , Chen S, Pireyre M, Glöckner N, Galvao VC, Albert A, Winkler JB, Fankhauser C, Harter K, Ulm R. 2018. *Arabidopsis* RUP2 represses UVR8-mediated flowering in noninductive photoperiods. Genes and Development 32, 1332–1343. 30254107 10.1101/gad.318592.118 PMC6169840

[CIT0002] Barnes PW , Robson TM, Neale PJ, et al. 2022. Environmental effects of stratospheric ozone depletion, UV radiation, and interactions with climate change: UNEP Environmental Effects Assessment Panel, Update 2021. Photochemical & Photobiological Sciences 21, 275–301. 35191005 10.1007/s43630-022-00176-5 PMC8860140

[CIT0003] Brown BA , Cloix C, Jiang GH, Kaiserli E, Herzyk P, Kliebenstein DJ, Jenkins GI. 2005. A UV-B-specific signaling component orchestrates plant UV protection. Proceedings of the National Academy of Sciences, USA 102, 18225–18230. 10.1073/pnas.0507187102 PMC1312397 16330762

[CIT0004] Camacho IS , Theisen A, Johannissen LO, Díaz-Ramos LA, Christie JM, Jenkins GI, Bellina B, Barran P, Jones AR. 2019. Native mass spectrometry reveals the conformational diversity of the UVR8 photoreceptor. Proceedings of the National Academy of Sciences, USA 116, 1116–1125. 10.1073/pnas.1813254116 PMC6347689 30610174

[CIT0005] Chen Z , Dong Y, Huang X. 2022. Plant responses to UV-B radiation: signaling, acclimation and stress tolerance. Stress Biology 2, 51. 37676395 10.1007/s44154-022-00076-9 PMC10441900

[CIT0006] Christie JM , Arvai AS, Baxter KJ, et al. 2012. Plant UVR8 photoreceptor senses UV-B by tryptophan-mediated disruption of cross-dimer salt bridges. Science 335, 1492–1496. 22323738 10.1126/science.1218091 PMC3505452

[CIT0007] Cloix C , Kaiserli E, Heilmann M, Baxter KJ, Brown BA, O’Hara A, Smith BO, Christie JM, Jenkins GI. 2012. C-terminal region of the UV-B photoreceptor UVR8 initiates signaling through interaction with the COP1 protein. Proceedings of the National Academy of Sciences, USA 109, 16366–16370. 10.1073/pnas.1210898109 PMC3479605 22988111

[CIT0008] Fang F , Lin L, Zhang QW, et al. 2022. Mechanisms of UV-B light-induced photoreceptor UVR8 nuclear localization dynamics. New Phytologist 236, 1824–1837. 36089828 10.1111/nph.18468 PMC9825989

[CIT0009] Favory JJ , Stec A, Gruber H, et al. 2009. Interaction of COP1 and UVR8 regulates UV-B-induced photomorphogenesis and stress acclimation in Arabidopsis. The EMBO Journal 28, 591–601. 19165148 10.1038/emboj.2009.4 PMC2657586

[CIT0010] Findlay KMW , Jenkins GI. 2016. Regulation of UVR8 photoreceptor dimer/monomer photo-equilibrium in Arabidopsis plants grown under photoperiodic conditions. Plant, Cell & Environment 39, 1706–1714. 10.1111/pce.12724 PMC5103188 26864532

[CIT0011] Gruber H , Heijde M, Heller W, Albert A, Seidlitz HK, Ulm R. 2010. Negative feedback regulation of UV-B-induced photomorphogenesis and stress acclimation in *Arabidopsis*. Proceedings of the National Academy of Sciences, USA 107, 20132–20137. 10.1073/pnas.0914532107 PMC2993346 21041653

[CIT0012] Hayes S , Velanis CN, Jenkins GI, Franklin KA. 2014. UV-B detected by the UVR8 photoreceptor antagonizes auxin signaling and plant shade avoidance. Proceedings of the National Academy of Sciences, USA 111, 11894–11899. 10.1073/pnas.1403052111 PMC4136589 25071218

[CIT0013] Heijde M , Binkert M, Yin RH, et al. 2013. Constitutively active UVR8 photoreceptor variant in Arabidopsis. Proceedings of the National Academy of Sciences, USA 110, 20326–20331. 10.1073/pnas.1314336110 PMC3864333 24277841

[CIT0014] Heijde M , Ulm R. 2013. Reversion of the UV-B photoreceptor UVR8 to the homodimeric ground state. Proceedings of the National Academy of Sciences, USA 110, 1113–1118. 10.1073/pnas.1214237110 PMC3549095 23277547

[CIT0015] Heilmann M , Christie JM, Kennis JT, Jenkins GI, Mathes T. 2015. Photoinduced transformation of UVR8 monitored by vibrational and fluorescence spectroscopy. Photochemical & Photobiological Sciences 14, 252–257. 25274012 10.1039/c4pp00246f

[CIT0016] Heilmann M , Velanis CN, Cloix C, Smith BO, Christie JM, Jenkins GI. 2016. Dimer/monomer status and function of salt-bridge mutants of the plant UV-B photoreceptor UVR8. The Plant Journal 88, 71–81. 27385642 10.1111/tpj.13260 PMC5091643

[CIT0017] Huang X , Ouyang XH, Yang PY, Lau OS, Chen LB, Wei N, Deng XW. 2013. Conversion from CUL4-based COP1–SPA E3 apparatus to UVR8–COP1–SPA complexes underlies a distinct biochemical function of COP1 under UV-B. Proceedings of the National Academy of Sciences, USA 110, 16669–16674. 10.1073/pnas.1316622110 PMC3799332 24067658

[CIT0018] Huang X , Yang PY, Ouyang XH, Chen LB, Deng XW. 2014. Photoactivated UVR8–COP1 module determines photomorphogenic UV-B signaling output in *Arabidopsis*. PLoS Genetics 10, e1004218. 24651064 10.1371/journal.pgen.1004218 PMC3961177

[CIT0019] Jenkins GI. 2014. The UV-B photoreceptor UVR8: from structure to physiology. The Plant Cell 26, 21–37. 24481075 10.1105/tpc.113.119446 PMC3963570

[CIT0020] Jenkins GI. 2017. Photomorphogenic responses to ultraviolet-B light. Plant, Cell & Environment 40, 2544–2557. 10.1111/pce.12934 28183154

[CIT0021] Jiang JJ , Liu J, Sanders D, Qian SM, Ren WD, Song JK, Liu FQ, Zhong XH. 2021. UVR8 interacts with de novo DNA methyltransferase and suppresses DNA methylation in *Arabidopsis*. Nature Plants 7, 184–197. 33495557 10.1038/s41477-020-00843-4 PMC7889724

[CIT0022] Kaiserli E , Jenkins GI. 2007. UV-B promotes rapid nuclear translocation of the UV-B-specific signaling component UVR8 and activates its function in the nucleus. The Plant Cell 19, 2662–2673. 17720867 10.1105/tpc.107.053330 PMC2002606

[CIT0023] Kliebenstein DJ , Lim JE, Landry LG, Last RL. 2002. Arabidopsis regulates ultraviolet-B signal transduction and tolerance and contains sequence similarity to human *Regulator of Chromatin Condensation 1*. Plant Physiology 130, 234–243. 12226503 10.1104/pp.005041 PMC166556

[CIT0024] Lau K , Podolec R, Chappuis R, Ulm R, Hothorn M. 2019. Plant photoreceptors and their signaling components compete for COP1 binding via VP peptide motifs. The EMBO Journal 38, e102140. 31304983 10.15252/embj.2019102140 PMC6745501

[CIT0025] Li XK , Liu ZY, Ren HS, Kundu M, Zhong FW, Wang LJ, Gao JL, Zhong DP. 2022. Dynamics and mechanism of dimer dissociation of photoreceptor UVR8. Nature Communications 13, 93. 10.1038/s41467-021-27756-w PMC8748919 35013256

[CIT0026] Liang T , Mei SL, Shi C, et al. 2018. UVR8 interacts with BES1 and BIM1 to regulate transcription and photomorphogenesis in *Arabidopsis*. Developmental Cell 44, 512–523.e5. 29398622 10.1016/j.devcel.2017.12.028

[CIT0027] Liao XY , Liu W, Yang HQ, Jenkins GI. 2020. A dynamic model of UVR8 photoreceptor signalling in UV-B-acclimated Arabidopsis. New Phytologist 227, 857–866. 32255498 10.1111/nph.16581

[CIT0028] Liao XY , Zhang B, Blatt MR, Jenkins GI. 2019. A FRET method for investigating dimer/monomer status and conformation of the UVR8 photoreceptor. Photochemical & Photobiological Sciences 18, 367–374. 30534791 10.1039/c8pp00489g PMC6374739

[CIT0029] Liu W , Giuriani G, Havlikova A, et al. 2024. Phosphorylation of Arabidopsis UVR8 photoreceptor modulates protein interactions and responses to UV-B radiation. Nature Communications 15, 1221. 10.1038/s41467-024-45575-7 PMC10858049 38336824

[CIT0030] Miyamori T , Nakasone Y, Hitomi K, Christie JM, Getzoff ED, Terazima M. 2015. Reaction dynamics of the UV-B photosensor UVR8. Photochemical & Photobiological Sciences 14, 995–1004. 25811405 10.1039/c5pp00012b

[CIT0031] Morales LO , Brosché M, Vainonen J, Jenkins GI, Wargent JJ, Sipari N, Strid A, Lindfors AV, Tegelberg R, Aphalo PJ. 2013. Multiple roles for UV RESISTANCE LOCUS8 in regulating gene expression and metabolite accumulation in Arabidopsis under solar ultraviolet radiation. Plant Physiology 161, 744–759. 23250626 10.1104/pp.112.211375 PMC3561016

[CIT0032] Podolec R , Demarsy E, Ulm R. 2021a. Perception and signaling of ultraviolet-B radiation in plants. Annual Review of Plant Biology 72, 793–822. 10.1146/annurev-arplant-050718-095946 33636992

[CIT0033] Podolec R , Lau K, Wagnon TB, Hothorn M, Ulm R. 2021b. A constitutively monomeric UVR8 photoreceptor confers enhanced UV-B photomorphogenesis. Proceedings of the National Academy of Sciences, USA 118, e2017284118. 10.1073/pnas.2017284118 PMC8017708 33542100

[CIT0034] Qian C , Chen Z, Liu Q, Mao W, Chen Y, Tian W, Liu Y, Han J, Ouyang X, Huang X. 2020. Coordinated transcriptional regulation by the UV-B photoreceptor and multiple transcription factors for plant UV-B responses. Molecular Plant 13, 777–792. 32126287 10.1016/j.molp.2020.02.015

[CIT0035] Qian CZ , Mao WW, Liu Y, Ren H, Lau OS, Ouyang XH, Huang X. 2016. Dual-source nuclear monomers of UV-B light receptor direct photomorphogenesis in *Arabidopsis*. Molecular Plant 9, 1671–1674. 27756574 10.1016/j.molp.2016.10.005

[CIT0036] Ren H , Han JP, Yang PY, et al. 2019. Two E3 ligases antagonistically regulate the UV-B response in *Arabidopsis*. Proceedings of the National Academy of Sciences, USA 116, 4722–4731. 10.1073/pnas.1816268116 PMC6410811 30787186

[CIT0037] Rizzini L , Favory JJ, Cloix C, et al. 2011. Perception of UV-B by the UVR8 protein. Science 332, 103–106. 21454788 10.1126/science.1200660

[CIT0038] Sharma A , Sharma B, Hayes S, Kerner K, Hoecker U, Jenkins GI, Franklin KA. 2019. UVR8 disrupts stabilisation of PIF5 by COP1 to inhibit plant stem elongation in sunlight. Nature Communications 10, 4417. 10.1038/s41467-019-12369-1 PMC6764944 31562307

[CIT0039] Shi C , Liu HT. 2021. How plants protect themselves from ultraviolet-B radiation stress. Plant Physiology 187, 1096–1103. 34734275 10.1093/plphys/kiab245 PMC8566272

[CIT0040] Wang LX , Wang YD, Chang HF, et al. 2023. RUP2 facilitates UVR8 redimerization via two interfaces. Plant Communications 4, 100428. 36065466 10.1016/j.xplc.2022.100428 PMC9860181

[CIT0041] Wang YD , Wang LX, Guan ZY, et al. 2022. Structural insight into UV-B-activated UVR8 bound to COP1. Science Advances 8, eabn3337. 35442727 10.1126/sciadv.abn3337 PMC9020657

[CIT0042] Wu D , Hu Q, Yan Z, et al. 2012. Structural basis of ultraviolet-B perception by UVR8. Nature 484, 214–219. 22388820 10.1038/nature10931

[CIT0043] Yang Y , Liang T, Zhang LB, Shao K, Gu XX, Shang RX, Shi N, Li X, Zhang P, Liu HT. 2018. UVR8 interacts with WRKY36 to regulate transcription and hypocotyl elongation in *Arabidopsis*. Nature Plants 4, 98–107. 29379156 10.1038/s41477-017-0099-0

[CIT0044] Yang Y , Zhang L, Chen P, Liang T, Li X, Liu H. 2020. UV-B photoreceptor UVR8 interacts with MYB73/MYB77 to regulate auxin responses and lateral root development. The EMBO Journal 39, e101928. 31777974 10.15252/embj.2019101928 PMC6960441

[CIT0045] Yin RH , Arongaus AB, Binkert M, Ulm R. 2015. Two distinct domains of the UVR8 photoreceptor interact with COP1 to initiate UV-B signaling in Arabidopsis. The Plant Cell 27, 202–213. 25627067 10.1105/tpc.114.133868 PMC4330580

[CIT0046] Yin RH , Skvortsova MY, Loubéry S, Ulm R. 2016. COP1 is required for UV-B-induced nuclear accumulation of the UVR8 photoreceptor. Proceedings of the National Academy of Sciences, USA 113, E4415–E4422. 10.1073/pnas.1607074113 PMC4968759 27407149

[CIT0047] Zeng XL , Ren Z, Wu Q, Fan J, Peng PP, Tang K, Zhang RQ, Zhao KH, Yang XJ. 2015. Dynamic crystallography reveals early signalling events in ultraviolet photoreceptor UVR8. Nature Plants 1, 14006. 26097745 10.1038/nplants.2014.6 PMC4469132

